# Downregulation of GluA2 AMPA Receptor Subunits Reduces the Dendritic Arborization of Developing Spinal Motoneurons

**DOI:** 10.1371/journal.pone.0049879

**Published:** 2012-11-30

**Authors:** Yone J. Yoon, Sheryl L. White, Xianglian Ni, Alexander P. Gokin, Miguel Martin-Caraballo

**Affiliations:** 1 Department of Biology, University of Vermont, Burlington, Vermont, United States of America; 2 Department of Anatomy and Neurobiology, College of Medicine, University of Vermont, Burlington, Vermont, United States of America; 3 Department of Pharmaceutical Sciences, School of Pharmacy, University of Maryland Eastern Shore, Princess Anne, Maryland, United States of America; Emory University, United States of America

## Abstract

AMPA receptors lacking the GluA2 subunit allow a significant influx of Ca^2+^ ions. Although Ca^2+^-permeable AMPA receptors are a familiar feature at early stages of development, the functional significance of these receptors during the maturation of the nervous system remains to be established. Chicken lumbar motoneurons express Ca^2+^-permeable AMPA receptors at E6 but the Ca^2+^ permeability of AMPA receptors decreases ∼3-fold by E11. Considering that activity-dependent changes in intracellular Ca^2+^ regulates dendritic outgrowth, in this study we investigated whether downregulation of GluA2 expression during a critical period of development alters the dendritic arborization of spinal motoneurons *in ovo*. We use an avian replication-competent retroviral vector RCASBP (B) carrying the marker red fluorescent protein (RFP) and a GluA2 RNAi construct to downregulate GluA2 expression. Chicken embryos were infected at E2 with one of the following constructs: RCASBP(B)-RFP, RCASBP(B)-RFP-scrambled RNAi, or RCASBP(B)-RFP-GluA2 RNAi. Infection of chicken embryos at E2 resulted in widespread expression of RFP throughout the spinal cord with ≥60% of *Islet1/2*-positive motoneurons infected, resulting in a significant reduction in GluA2 protein expression. Downregulation of GluA2 expression had no effect on the dendritic arborization of E6 motoneurons. However, downregulation of GluA2 expression caused a significant reduction in the dendritic arborization of E11 motoneurons. Neither motoneuron survival nor maturation of network activity was affected by changes in GluA2 expression. These findings demonstrate that increased GluA2 expression and changes in the Ca^2+^ permeability of AMPA receptors regulate the dendritic arborization of spinal cord motoneurons during a critical period of development.

## Introduction

Dendritic outgrowth is a critical aspect of network formation which allows the establishment of appropriate synaptic connections with other neuronal components. Dendrites are required to navigate complex environments in order to make appropriate connections with their targets. Dendritic outgrowth is a highly dynamic process that depends both on intrinsic factors and extrinsic interactions with other network components [1], [2], [3], [4], [5]. Dendritic morphology will ultimately determine the number and location of synaptic inputs, which in term regulate the ability of neurons to integrate and process afferent information. Understanding how dendritic morphology is regulated at early stages of development is critical in order to gain a better understanding of the cellular and molecular mechanisms involved in network formation.

Using the chicken spinal cord as a model, we have previously demonstrated that the dendritic arborization of spinal motoneurons undergo considerable changes during a critical period of development spanning from embryonic day (E) 6 to E11 [6]. During this period, there is a dramatic increase in the complexity of the dendritic tree of motoneurons as evidenced by an increase in dendritic length, the number of branch points and dendritic ends. Functionally, maturation of the dendritic morphology of spinal motoneurons between E6 and E11 is critical for the establishment of synaptic contacts with sensory afferents and descending supraspinal inputs at E8, which regulate the generation of spinal cord reflexes and locomotor activity, respectively [7], [8]. The cellular mechanisms involved in the maturation of dendritic morphology of motoneurons during this period of development remains to be established.

Activation of Ca^2+^-permeable glutamate receptors constitutes one important source of Ca^2+^ entry in developing neurons. Although NMDA receptors were originally thought to be a main source of Ca^2+^ influx through glutamate receptors, AMPA receptors lacking the GluA2 subunit (IUPHAR nomenclature, [9]) also have a significant Ca^2+^ permeability [10], [11]. Chicken spinal motoneurons express Ca^2+^-permeable AMPA receptors at embryonic day (E) 6, however, the Ca^2+^ permeability of AMPA receptors decreases ∼3-fold by E11 [12]. The switch in the Ca^2+^ permeability of AMPA receptors is triggered by a significant increase in GluA2 expression by E8 without any changes in the editing pattern of GluA2 subunits [12]. It appears that activation of Ca^2+^-permeable AMPA receptors regulates the maturation of the dendritic morphology of developing motoneurons during a critical period of development. For example, pharmacological inhibition of Ca^2+^-permeable AMPA receptors between E6 and E8 evokes a significant increase in the dendritic arborization of motoneurons [6]. In this study, we investigated whether changes in GluA2 expression regulate the dendritic morphology of developing motoneurons by directly altering the Ca^2+^-permeability of AMPA receptors or by evoking changes in network activity. We used an RCASBP(B) viral construct containing a GluA2 RNAi insert in order to downregulate GluA2 expression in the developing spinal cord.

## Methods

### RCASBP (B) gene construct and virus production

In order to knock down the chicken GluA2 levels *in ovo*, short interfering RNAs (siRNAs) were made against specific regions of the GluA2 transcript. Initially, three different siRNAs were prepared against 3 different regions of the GluA2 transcript (GluA2 siRNA1 target sequence: ggagctctccttagtttgattg; GluA2 siRNA2 target sequence: atattggagtgaagtggacaaa; and GluA2 siRNA3 target sequence: tggctttgattgagttctgtta). The oligonucleotides used for these regions were selected using the Whitehead Institutes siRNA selection server [13]. Following PCR to form the siRNA with hairpin primers, the siRNA PCR fragment was then cloned into the miRNA30-like hairpin insertion site in a slx12 shuttle vector containing RFP, along with a U6 siRNA cassette containing the chicken U6 promoter and a microRNA operon expression cassette. Following sequence confirmation of the microRNA cassette that the desired siRNA had been properly inserted, the entire Cla1 fragment consisting of the chicken U6-microRNA expression cassette with GluA2 siRNA and RFP was cloned into the Cla1 site of the retroviral plasmid RCASBP(B) [14]. The RCASBP(B) constructs were then transfected via liposome (Mirus Bio, Madison, WI) into a chicken fibroblast cell line (DF-1 cells, American Type Culture Collection, Manassas, VA) in order to produce infective RCASBP(B)-RFP-GluA2 siRNA retroviruses. DF-1 cells cultures were maintained in modified L15 medium supplemented with 10% heat-inactivated horse serum, 50 U/ml penicillin, and 50 µg/ml streptomycin. Concentration of viral stocks was performed by ultracentrifugation at 90,000 g at 4°C for 3 hr. After determining viral titers (>10^8^ infectious particles/mL), constructs were aliquoted and stored at −80°C until use.

The retroviruses containing the GluA2 siRNA cassette were then used to infect the chicken embryo targets. This approach allows the visual identification by epifluorescence microscopy of infected cells containing the RFP, in conjunction with the expression of the GluA2 siRNA. Initial experiments demonstrated that the first GluA2 siRNA1 target sequence was the most effective in downregulating GluA2 expression in the chicken spinal cord, accordingly, this sequence was the one used for this study and is represented throughout the text as RCASBP(B)-RFP-GluA2 siRNA. An additional negative control was also constructed, a scrambled oligonucleotide of the same length and residue content as the GluA2 siRNA oligonucleotides but with no known chicken target, which was also cloned into the RCASBP(B) vector (scrambled siRNA sequence: tggacataggcgacgtgat). The RCASBP(B)-RFP-scrambled siRNA construct was used to generate retrovirus used for infection of the chicken embryos in the same manner as the RCASBP(B)-RFP-GluA2 siRNA construct.

### Viral Infections

Viral infection of chicken embryos was performed as previously described by Yoon et al. [15]. Pathogen-free eggs were obtained from SPAFAS (Charles River Laboratories, Wilmington, MA) and incubated at 37°C. Embryos were staged according to Hamburger and Hamilton [16]. Prior to viral injections, a small window was cut in the shell directly above the embryo. Concentrated viral stocks were injected into the neural tube of E2 chicken embryos (corresponding to stage 8–10) using a fine tip pipette. In the chicken embryo, most lumbar motoneurons become postmitotic by E4 [17]. After the viral injections, the window in the egg's shell was closed with Scotch tape (3 M, St. Paul, MN) and embryos were returned to the incubator. Embryos were incubated in a humidified incubator at 37°C until E6 (corresponding to stages 24–26), E8 (stage 33–34), or E11 (corresponding to stage 37). No gross morphological differences were observed between controls (non-injected) or embryos infected with the RCASBP(B) constructs. We should point out that in this study we only included in our analysis embryos with extensive rostro-caudal and dorso-ventral RFP labeling of the lumbar section of the spinal cord. Embryos with only partial labeling of the lumbar spinal cord were not included in this study.

### Western blot analysis

For immunoblot analysis of GluA2 protein expression, ventral spinal cords were isolated in ice-cold Ca^2+^/Mg^2+^-free saline and lysed in RIPA buffer supplemented with a protease inhibitor cocktail (Sigma) as previously reported by Ni et al. [12]. After determining the protein concentration with a Bradford protein kit (BioRad, Hercules, CA), lysates were combined with 2× Laemmli sample buffer and boiled for 5 min at 95°C. Samples were separated by SDS-PAGE on 8% gels. Proteins were transferred to PVDF membranes (Millipore, MA). After transfer, membranes were blocked with Aquablock blocking buffer (EastCoastBio, North Berwick, ME) overnight at 4°C. Membranes were incubated with a mouse anti-GluA2 antibody (1∶500 dilution, Chemicon, Temecula, CA) dissolved in blocking buffer for 4 hr. After four washes, membranes were incubated with a 1∶10,000 dilution of the IRDye 800 anti-rabbit IgG (Rockland, Gilbertsville, PA) secondary antibody in the dark. After washing the membranes three times, an image of the IRDye 800 signal was acquired using the 800 nm channel of the Odyssey infrared imaging system (LICOR Biosciences GmbH, Lincoln, NE). Following detection of GluA2 expression, membranes were stripped in a glycine-based solution and reprobed with a mouse anti-β-actin antibody (at 1∶20,000 dilution, Sigma) in order to asses GluA2 protein expression as a function of total protein content in each well. This was followed by incubation with the corresponding secondary antibody (IRDye 800 anti-rabbit IgG) and immunodetection. Each experiment was repeated 3–4 times.

### Assessment of dendritic morphology

Dendritic morphology was assessed as previously described by Ni and Martin-Caraballo [6] and Yoon et al. [18]. Embryos were fixed in 4% paraformaldehyde in phosphate buffered saline (PBS) overnight. After fixation for 24 hr, a small amount of DiI (2–4 µL) was applied using a picospritzer (Parker, Fairfield, NJ) onto the nerves in the ischiadic plexus, and embryos were returned to the incubator for up to 8 weeks to allow time for complete neuronal labeling. Motoneurons of the ischiadic plexus can be found throughout lumbar segments L3 to L8 [19]. The entire lumbar portion of the spinal cord was isolated (L1–L8) and sectioned into 200 µm slides. The use of thicker sections (200 µm) allowed us to obtain labeled motoneurons with their transverse dendritic tree intact (at least in the cross sectional plane). Therefore, our present analysis of dendritic morphology refers only to a portion of the dendritic tree, which should be taken into consideration when interpreting the data. Analysis of dendritic morphology was performed in 4–5 spinal cords per group. After sectioning of the entire lumbar segments into 200 µm slides, all sections were examined for the presence of labeled motoneurons. Only sections containing 1–3 labeled motoneurons were used for tracing of dendritic arborization. Spinal cord sections with >3 labeled motoneurons were not analyzed because it was difficult to trace the dendritic tree under those conditions.

Labeled motoneurons were imaged with a Nikon Eclipse E600 microscope. Tracing of individually labeled motoneurons was performed using a computer-assisted camera morphometric program (Neurolucida, Microbrightfield, Colchester VT). Three criteria were used in selecting appropriate motoneurons for tracing [6], [18]. First, only motoneurons with intact dendrites on the cross-sectional plane of the 200 µm section were included in our analysis (we did not analyze dendrites extending in the rostro-caudal direction, see below). Second, only motoneurons sufficiently separated from their neighbors were used for tracing. We have determined that the key to successful tracing is to apply a small amount of DiI in each nerve, which will only result in the labeling of at most 3 motoneurons/section. Extensive application of DiI resulted in labeling of a large number of neurons, which hindered the visualization and tracing processes of single motoneurons. Third, only motoneurons with primary dendrites emanating >180 degrees from the cell body were considered. This was an indication that DiI has spread evenly in all directions within the cell. In each DiI-labeled motoneuron, the following parameters were measured: dendritic arbor/cell, the longest dendritic tree derived from a primary dendrite (represented as the longest dendrite), number of primary dendrites, number of nodes (branch points), and number of ends. We should mention that our technique for assessing dendritic arborization only included dendrites located in the transverse plane of the spinal cord but obviously did not include dendrites extending in the rostro-caudal direction. Therefore, our definition of dendritic arbor/cell refers to the length of all dendritic segments lying exclusively in the transverse plane of the spinal cord section. To investigate whether changes in the dendritic tree are localized to a particular area, we assessed dendritic length and/or number according to branch order. In this analysis, we compared the total length of primary dendrites, followed by second order dendrites (or dendrites bifurcating from primary dendrites) and so on. This allowed us to determine whether any changes in dendritic length may occur in proximal dendrites or more distal dendrites. Changes in soma morphology were assessed by measuring cell body perimeter and somatic surface area. The cell body perimeter was measured by focusing on the plane of the cell body, where cell dimensions were the greatest, and outlining the cell body contour.

### Motoneuron dissociation and cell culture

Isolation of lumbar motoneurons was performed as previously described by Martin-Caraballo and Dryer [20]. For an enriched motoneuron culture, only the ventral sections of the chicken spinal cord were excised into a Ca^2+^/Mg^2+^-free solution, mildly trypsinized (E6, 0.05% for 20 min; E8, 0.05% for 30 min; E11, 0.2% for 40 min), dissociated by trituration, and plated onto poly-d-lysine-coated glass coverslips. Basal culture medium consisted of Eagle's minimal essential medium (EMEM, BioWhittaker, Walkersville, MA) supplemented with 10% heat-inactivated horse serum, 2 mM glutamine, 50 U/ml penicillin, 50 µg/ml streptomycin, and 10 ng/mL glial derived neurotrophic factor (GDNF). Recording of whole-cell currents was performed following overnight culture.

### *Islet1/2* immunohistochemistry and design-based stereology

Counting of the number of *Islet1/2*-positive neurons was performed as previously described by Yoon et al. [15], [18]. Six segments of the lumbar enlargement (L1–L6) were removed at E10 and fixed in Zamboni's fixative [4% paraformaldehyde +15% picric acid in 0.1 M phosphate buffer, pH = 7.4] at 4°C overnight, washed three times in phosphate buffer saline (PBS), and equilibrated in 30% sucrose/PBS overnight. Spinal cord tissue was embedded in OCT freezing medium, and 30 µm-cryostat sections were serially collected using a Leica cryostat. Sections were air dried for 5 min and postfixed in 4% paraformaldehyde for 30 min. Slides were washed three times in 0.1 M PBS and blocked overnight in blocking solution (PBS containing 10% horse serum and 0.5% Triton X-100) at 4°C. Sections were incubated overnight at 4°C with an anti-*Islet1/2* (1∶100 mouse hybridoma supernatant, clone 39.405, Developmental Studies Hybridoma Bank, University of Iowa) diluted in blocking solution. This antibody recognizes expression of both *Islet* 1 and 2 [21], [22]. Following three washes with PBS, sections were incubated with 0.5% hydrogen peroxide for 30 min to block endogenous peroxidase activity. After three more washes with PBS, slides were incubated for 2 hr at room temperature with a biotinylated goat anti-mouse antibody (1∶500, Vector Laboratories). Following three washes with PBS, slides were incubated with Vectastain ABC-HRP solution for 3–4 hr at room temperature. *Islet1/2* staining was visualized by using a nickel/cobalt enhanced diaminobenzidine solution. After three washes, slides were mounted using AquaMount (Lerner Laboratories, Pittsburgh, PA). The number of *Islet1/2*-positive neurons on both sides of the ventral spinal cord was counted in every fifth section using StereoInvestigator software (Microbrightfield Inc, Williston, VT). Images were obtained with a Nikon Eclipse E600W microscope coupled to a MicroFire video camera (Optronics) and with an x,y,z stage drive and position transducer (MAC 2000, Ludl Electronic Products, Ltd.). Under low magnification, the boundary of the motoneuron pool was identified and the boundary contour was drawn using the software-pointing device. A randomly generated sampling grid was placed over the contour area, containing 5–10 square counting frames (175×175 µm). Only *Islet1/2*-stained nuclei within the counting frame and no contact with exclusion lines were counted using a 40× objective. The total number of motoneurons was obtained by adding together all counted neurons along L1–L6 spinal segments and multiplying by five.

### *Islet1/2* and p27 gag immunocytochemistry

Double staining of cultured spinal cord neurons with the motoneuron marker *Islet1/2* and the RCASBP viral protein marker p27 gag was performed as previously described by Yoon et al. [15]. Briefly, cultures of isolated ventral spinal cord neurons were fixed in Zamboni's fixative and blocked in blocking solution for 1 hr at room temperature. Cells were then incubated overnight with various primary antibodies (mouse anti-*Islet1/2* at 1∶250 or rabbit anti-p27 gag at 1∶2000) in blocking solution at 4°C. After three washes, sections were incubated for 1 h with the corresponding secondary antibodies (Alexa 488-conjugated anti-mouse and Cy3-conjugated anti-rabbit diluted at 1∶750, respectively). Cells were mounted in VectaShield medium (Vector Labs, Burlingame, CA) and visualized using a Nikon fluorescent microscope.

### Electrophysiology

Dissociated motoneurons were identified during patch-clamp recordings using an Olympus X71 inverted microscope equipped with Hoffman optics and rhodamine filters. Recordings were performed at room temperature (22–24°C). Recording electrodes were made from thin wall borosilicate glass (3–4 MΩ) and filled with a solution consisting of (in mM): 120 Cs aspartate, 2 MgCl_2_, 10 HEPES, 10 EGTA, 1 ATP, and 0.1 GTP (pH 7.4 with CsOH). To investigate the Ca^2+^ permeability of AMPA receptors, cell cultures were perfused with an external solution in which NaCl was replaced with the impermeant cation N-methylglucamine (NMG), and 10 mM CaCl_2_ as previously reported by Ni et al. [12]. The composition of the 10 mM Ca^2+^/Na^+^-free extracellular solution was (in mM): 135 NMG, 10 CaCl_2_, 5 glucose, and 10 HEPES (pH 7.4 with HCl). Under these recording conditions, kainate currents are mediated by the flow of Ca^2+^ and Cs^+^ ions. The permeability ratio (P_Ca_/P_Cs_) in the 10 mM Ca^2+^/Na^+^-free solution was calculated from the reversal potential (E_r_) according to the extended GHK constant field equation using estimated ion activities [23]: P_Ca_/P_Cs_ = 0.25×(a_Cs_/a_Ca_)×exp (E_r_F/RT)×[exp (E_r_F/RT)+1], where a_Cs_ = Cs^+^ activity (activity coefficient = 0.75), a_Ca_ = Ca^2+^ activity (activity coefficient = 0.55), and F, R, and T have their usual meaning. All E_r_ values were adjusted for an estimated junction potential of 10.2 mV (in 10 mM Ca^2+^/Na^+^-free solution). Drugs were applied using a gravity-fed perfusion system (Bioscience Tools, San Diego, CA). Voltage commands and data acquisition and analysis were performed with a MultiClamp 700A amplifier and Pclamp software (Axon Instruments, Foster City, CA). Pipette offset and whole cell capacitance were compensated automatically with the MultiClamp 700B Commander.

### Extracellular recordings of spinal cord activity

Recording of spontaneous electrical activity was performed as previously described by Yoon et al. [18]. Briefly, chicken embryos were isolated at E11 and the lumbar spinal cord was dissected in a cool (15°C) oxygenated Tyrode's solution supplemented with 12 mM glucose. After dissection, the spinal cord was transferred to a recording chamber and kept overnight while perfusing with cool (17°C) oxygenated Tyrode's solution. The following morning, the spinal cord was warmed for 1 hr by perfusing with Tyrode's solution at room temperature. After 1 hr, the temperature of the preparation was raised again to 27°C in order to induce the generation of spontaneous network activity. Spinal cord activity was recorded using an extracellular electrode inserted in the motoneuron pool. Electrodes with 4–5 MΩ resistance were filled with a 145 mM NaCl solution. Extracellular activity was recorded with an Axon patch amplifier after compensation of pipette junction potentials.

### Data Analysis

Values are presented as mean ± SEM where indicated. Statistical analyses consisted of one-way ANOVA followed by *post hoc* analysis using Tukey's honest significant difference test for unequal *n* for comparisons between multiple groups (SigmaStat software). Throughout, *p*≤0.05 was regarded as significant.

## Results

**Figure 1:**
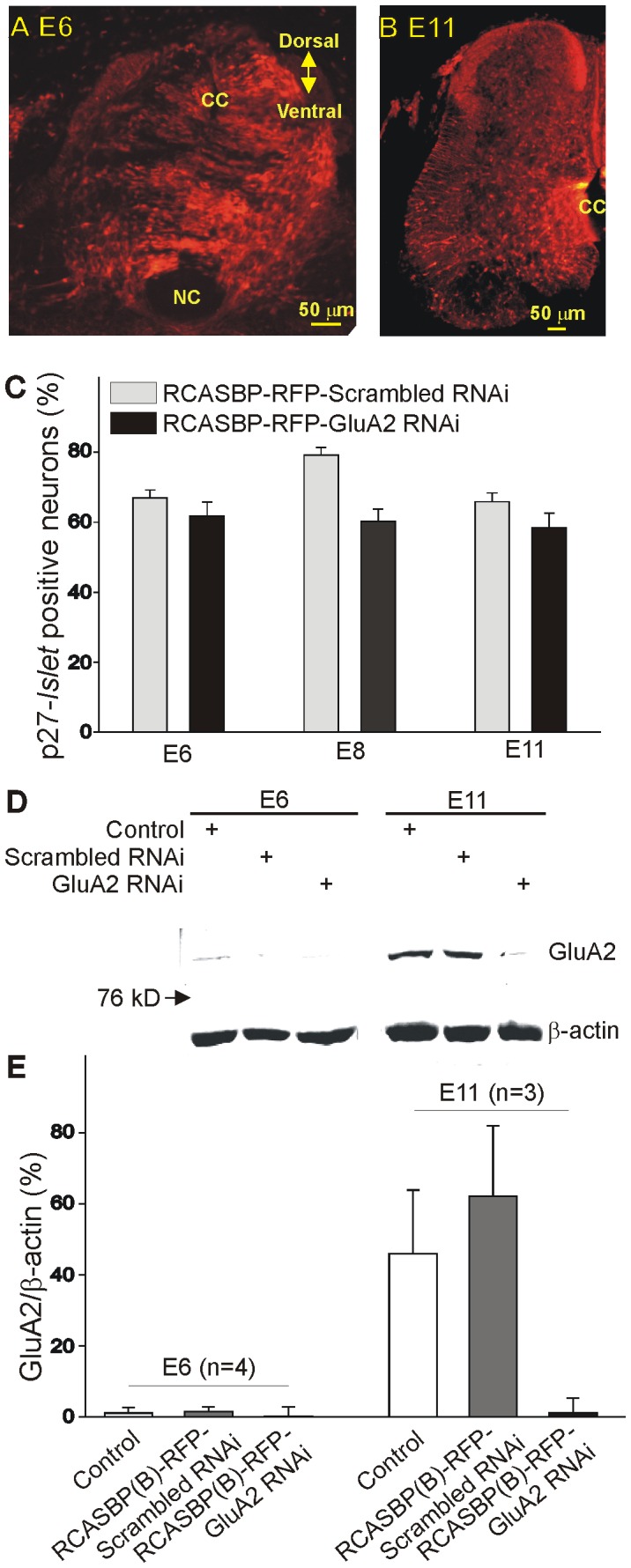
Effect of RCASBP(B)-RFP-GluA2 RNAi infection on chicken embryos. ***A–B*** *)* Expression of red fluorescence protein (RFP) transgene in the lumbar spinal cord of E6 (***A***) and E11 (***B***) chicken embryos following retroviral infection with an RCASBP(B)-RFP-GluA2 RNAi construct. Embryos infected with the RCASBP(B)-RFP-GluA2 RNAi construct show strong fluorescent labeling throughout the whole spinal cord cross section. cc = central canal, nc = notochord. ***C*** *)* Averaged number of labeled neurons for the RCASBP(B) viral protein p27 gag as a percent of the total number of neuron labeled with the motoneuron marker *Islet1/2* in chicken embryos infected with an RCASBP(B)-RFP-scrambled RNAi or RCASBP(B)-RFP- GluA2 RNAi construct. Lumbar ventral neurons were isolated from E6, E8 or E11 chicken embryos and immunolabeled with p27 gag and *Islet1/2* in order to assess the extent of viral infection of spinal motoneurons. Notice that ≥60% of infected cells also tested positive for the motoneuron marker *Islet1/2* at all ages tested. ***D*** *)* Representative example of Western Blot data collected from the E6 and E11 ventral spinal cords from control chicken embryos (non-infected) or embryos infected with an RCASBP(B)-RFP-scrambled RNAi or RCASBP(B)-RFP-GluA2 RNAi constructs. The anti-GluA2 antibody detected a band with a relative molecular weight of ∼102 kD. To normalize for changes in protein loading in each well, membranes were reprobed for β-actin (∼42 kD). Infection of chicken embryos with an RCASBP(B)-RFP- GluA2 RNAi construct causes a significant reduction in GluA2 expression at E11 as determined by immunoblot analysis. ***E*** *)* Expression of GluA2 protein as a function of β-actin in chicken ventral spinal cords. The age-dependent increase in GluA2 protein expression between E6 and E11 chicken spinal cords was reversed by infection of chicken embryos with an RCASBP(B)-RFP- GluA2 RNAi construct. In these experiments, RCASBP(B)-RFP-scrambled RNAi or RCASBP(B)-RFP-GluA2 RNAi viral particles were injected into the developing neural tube at E2 (approximately 36 hr after incubation). Embryos were allowed to develop up to E11 before tissue isolation and processing.

As previously reported, GluA2 expression in the chicken spinal cord becomes significant after E8 [12]. In order to downregulate GluA2 expression, chicken embryos were infected with an RCASBP(B)-RFP-GluA2 siRNA construct targeting a specific region of the chicken GluA2 transcript. The RCASBP(B)-RFP-GluA2 siRNA construct was injected into the chicken neural tube at E2. At this stage, motoneuron precursors are still dividing [17]. Controls consisted of chicken embryos infected with an RCASBP(B)-RFP-scrambled siRNA or the RCASBP(B)-RFP open vector. All RCASBP(B) constructs used also contained the RFP gene, which was used to assess the extent of viral infection of spinal neurons. After viral infections, embryos were allowed to develop up to E11. Spinal cords were isolated at E6, E8 and E11 in order to assess viral transfection and RFP expression. Infection of chicken embryos with the RCASBP(B)-RFP- GluA2 siRNA construct resulted in a significant expression of RFP throughout the whole spinal cord in E6 and E11 chicken embryos (Fig. 1A–B). To assess the extent of viral infection of spinal motoneurons, we performed double labeling of cultured cells with the motoneuron marker *Islet1/2* and the viral protein p27 as previously reported [15]. Non-infected embryos did not show any labeling for the viral gag p27 protein (not shown). As represented in Fig. 1C, ≥60% of infected cells were also *Islet1/2-*positive neurons at all ages tested, suggesting that a majority of spinal motoneurons become infected with the viral constructs RCASBP(B)-RFP-scrambled siRNA and RCASBP(B)-RFP-GluA2 siRNA.

In order to assess whether the RCASBP(B)-RFP-GluA2 siRNA construct is effective in decreasing GluA2 expression in the chicken spinal cord, we performed western blot analysis using samples of the ventral spinal cord of chicken embryos at E6 and E11 (Fig. 1D–E). Consistent with our previously published results [12], immunoblot analysis demonstrates an increasing pattern of GluA2 protein expression in the chicken spinal cord between E6 and E11 (Fig. 1D). Infection of chicken embryos with the RCASBP(B)-RFP-GluA2 siRNA construct has no effect of the GluA2 protein levels at E6 since at this developmental stage GluA2 expression is minimal (Fig. 1D, [12]). However, in RCASBP(B)-RFP-GluA2 siRNA-infected embryos isolated at E11, GluA2 protein expression is significantly reduced (Fig. 1D). In order to quantify changes in GluA2 expression as a function of protein levels in each sample, membranes were reprobed for β-actin. As indicated in Fig. 1E, the ratio of GluA2 to β-actin was significantly low at E6. The GluA2/β-actin ratio increased significantly by E11 in non-infected or RCASBP(B)-RFP- scrambled siRNA-infected embryos. Infection of chicken embryos with the RCASBP(B)-RFP-GluA2 siRNA construct leads to a significant reduction in the GluA2/β-actin ratio when compared with non-infected or RCASBP(B)-RFP-scrambled siRNA-infected embryos (Fig. 1E).

**Figure 2:**
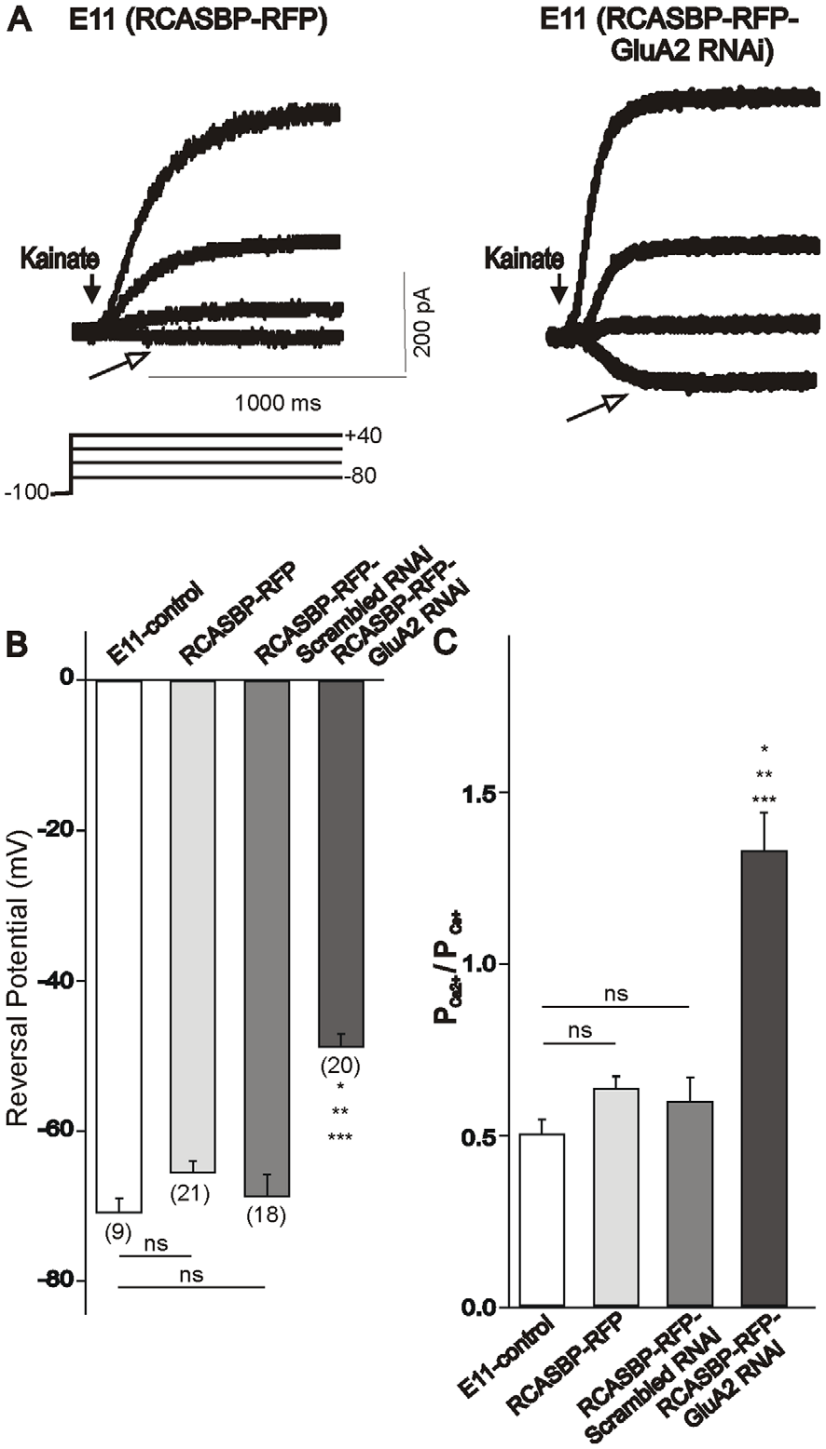
Changes in the Ca^2+^-permeability of AMPA receptors evoked by infection of chicken embryos with an RCASBP(B)-RFP-GluA2 RNAi contruct. ***A*** *)* Typical whole-cell currents in E11 motoneurons evoked by kainate application in 10 mM Ca^2+^/Na^+^-free external solution, at four different holding potentials ranging from −80 to +40 mV. Notice that in motoneurons infected with the RCASB(B)-RFP-GluA2 RNAi viral construct there is a significant increase in the inward Ca^2+^ currents evoked by kainate (empty arrow) at a holding potential of −80 mV. **B**) Plot of the reversal potential of kainate-evoked currents obtained in E11 motoneurons isolated from non-infected embryos (control) or embryos infected with RCASBP(B)-RFP, RCASBP(B)-RFP-scrambled RNAi or RCASBP(B)-RFP-GluA2 RNAi viral particles. **C**) Plot showing the relative permeability of Ca^2+^ to that of Cs^+^ (P_Ca2+_/P_Cs+_) in E11 motoneurons isolated from non-infected embryos or embryos infected with RCASBP(B)-RFP, RCASBP(B)-RFP-scrambled RNAi or RCASBP(B)-RFP-GluA2 RNAi viral particles. The ratio of P_Ca2+_/P_Cs+_ was calculated according to the extended GHK constant field equation (see Methods). Infection of chicken embryos with an RCASBP(B)-RFP-GluA2 RNAi construct results in a significant increase in the Ca^2+^ permeability of kainate-activated channels compared with control, RCASBP(B)-RFP or RCASBP(B)-RFP-scrambled RNAi-infected embryos (*p≤0.05 vs. control, **p≤0.05 vs. RCASBP(B)-RFP, ***p≤0.05 vs. RCASBP(B)-RFP-scrambled RNAi).

Our previous findings indicate that between E6 and E11 there is a ∼3-fold reduction in the relative Ca^2+^ permeability of AMPA receptors of spinal motoneurons due to a significant increase in GluA2 expression [12]. Thus, the question arises: does downregulation of GluA2 expression with the RCASBP(B)-RFP-GluA2 siRNA construct in spinal motoneurons result in the functional expression of Ca^2+^-permeable AMPA receptors at E11? To determine whether infection of spinal motoneurons with the RCASBP(B)-RFP-GluA2 siRNA construct leads to a significant increase in the Ca^2+^ permeability of AMPA receptors in E11 motoneurons, we recorded the kainate-generated currents in a Na^+^-free, high Ca^2+^ extracellular solution as previously reported [12]. Kainate-evoked currents were recorded from RFP-positive neurons. Membrane potential was held at −100 mV and depolarizing pulses were applied to potential between −80 and +40 mV. An example of kainate-generated currents in E11 motoneurons from RCASBP(B)-RFP and RCASBP(B)-RFP-GluA2 siRNA-infected embryos is represented in Fig. 2A. Kainate evokes a significant inward current at very negative membrane potentials (−80 mV) in motoneurons isolated from RCASBP(B)-RFP-GluA2 siRNA-infected embryos but not from embryos infected with the RCASBP(B)-RFP construct. In order to determine the relative Ca^2+^ permeability of AMPA receptors we measure the reversal potential of the kainate-generated currents under our recording conditions. The reversal potentials of kainate-generated currents in motoneurons from RCASBP(B)-RFP-GluA2 siRNA-infected embryos were significantly more positive than those from non-infected, RCASBP(B)-RFP or RCASBP(B)-RFP-scrambled siRNA-infected embryos (Fig. 2B). Changes in the P_Ca_/P_Cs_ were assessed using the constant field equation (see Methods) based on the reversal potential of kainate-generated currents in the 10 mM Ca^2+^/Na^+^-free extracellular solution (Fig. 2C, [12]). There were no significant differences in the P_Ca_/P_Cs_ between non-infected and RCASBP(B)-RFP-infected embryos (Fig. 2C). However, in motoneurons isolated from RCASBP(B)-RFP-GluA2 siRNA-infected embryos there was a significant increase in the relative Ca^2+^ permeability of AMPA receptors as indicated by a 50% increase in P_Ca_/P_Cs_ ratio (Fig. 2C). These results suggest that infection of spinal motoneurons with the RCASBP(B)-RFP-GluA2 siRNA construct leads to a significant increase in the relative Ca^2+^ permeability of AMPA receptors in E11 motoneurons that is similar to that seen in E6 motoneurons [12].

**Figure 3:**
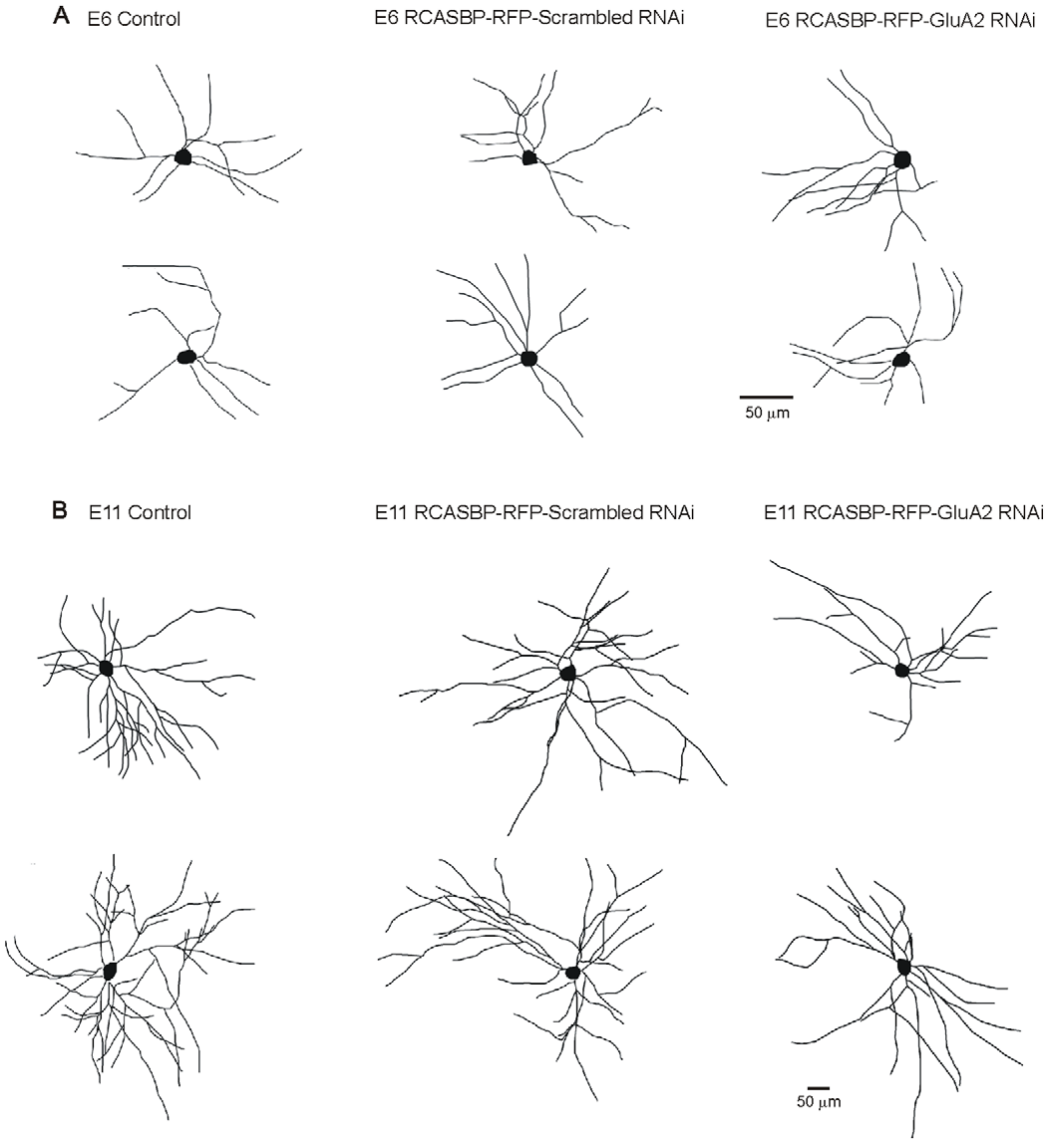
Typical Neurolucida drawing of DiI-labeled motoneurons, traced from control (non-infected) or RCASBP(B)-RFP-scrambled RNAi or RCASBP(B)-RFP-GluA2 RNAi-infected chicken embryos. The dendritic morphology of the motoneurons was assessed at E6 (*A*) or E11 (*B*). There are little changes in the architecture of the dendritic tree of E6 motoneurons following infection with the RCASBP(B)-RFP-GluA2 RNAi vital construct when compared with control or RCASBP(B)-RFP-scrambled RNAi-infected embryos. However, there is a significant reduction in the complexity of the dendritic tree of E11 motoneurons following infection with the RCASBP(B)-RFP-GluA2 RNAi viral construct (the dendritic tree appears less complex and with fewer branches).

**Figure 4:**
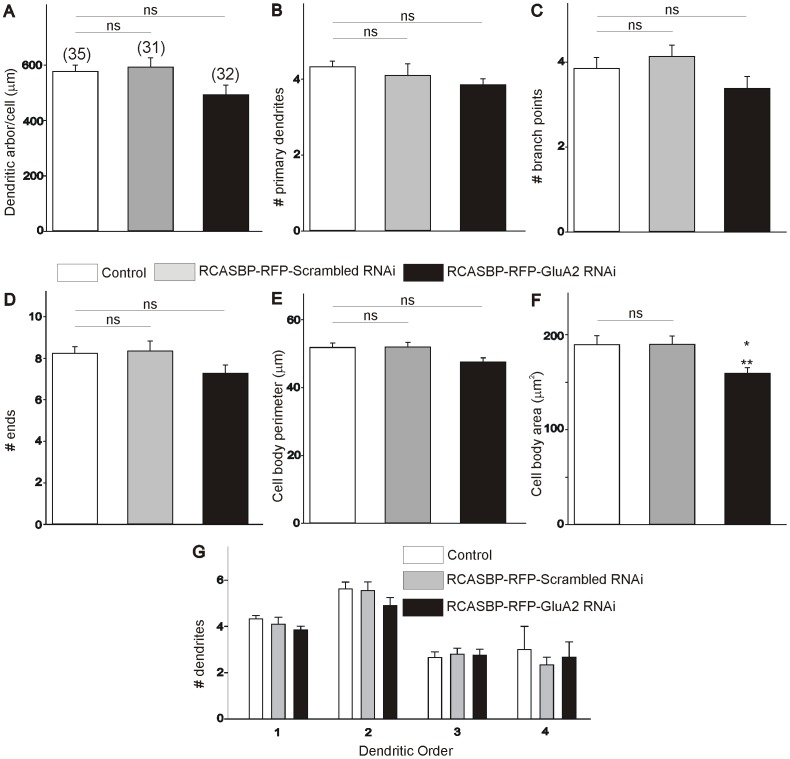
Effect of GluA2 RNAi on the dendritic morphology of E6 chicken lumbar motoneurons. ***A–D*** *)* Infection of chicken embryos with the RCASBP(B)-RFP-GluA2 RNAi construct has no effect on the dendritic complexity of E6 motoneurons including dendritic arbor/cell (***A***), number of primary dendrites (***B***), number of branches (*C*), and number of ends (***D***). ***E–F***) Infection of chicken embryos with the RCASBP(B)-RFP-GluA2 RNAi construct has no effect on the cell body perimeter (***E***) but causes a significant reduction in soma area (***F***). ***G***) Comparison of E6 dendritic morphology as a function of dendritic order in control embryos or embryos infected with an RCASBP(B)-RFP-scrambled RNAi or RCASBP(B)-RFP-GluA2 RNAi construct.

**Figure 5:**
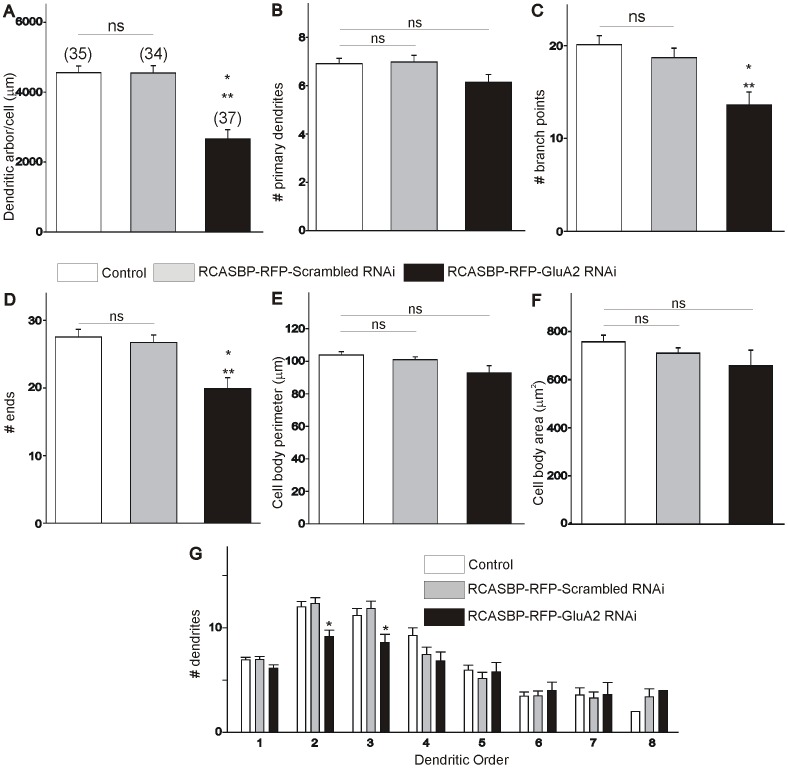
Effect of GluA2 RNAi on the dendritic morphology of E11 chicken lumbar motoneurons. *A* ***–D*** *)* Infection of chicken embryos with the RCASBP(B)-RFP-GluA2 RNAi construct causes a significant decrease in the dendritic complexity of E11 motoneurons. There is a significant reduction in the overall length of the dendritic arbor/cell (***A***), number of primary dendrites (***B***), number of branch points (***C***), and number of ends (***D***). ***E–F*** *)* Infection of chicken embryos with the RCASBP(B)-RFP-GluA2 RNAi construct has no effect on the cell body morphology of E11 motoneurons including cell body perimeter (***E***) and cell body area (***F***). ***G***) Comparison of E11 dendritic morphology as a function of dendritic order in control embryos and RCASBP(B)-RFP-scrambled RNAi or RCASBP(B)-RFP-GluA2 RNAi–infected embryos. Downregulation of GluA2 expression causes a significant reduction of the number of proximal dendrites (2^nd^ and 3^rd^ dendritic orders) and no effect on higher order dendrites (4^th^–8^th^ dendritic orders) when compared to control or RCASBP(B)-RFP-scrambled RNAi-infected embryos. * p<0.05 vs. control; ** p<0.05 vs. RCASBP(B)-RFP-scrambled RNAi. ns denotes no significant differences between the groups as indicated by one-way ANOVA.

**Figure 6:**
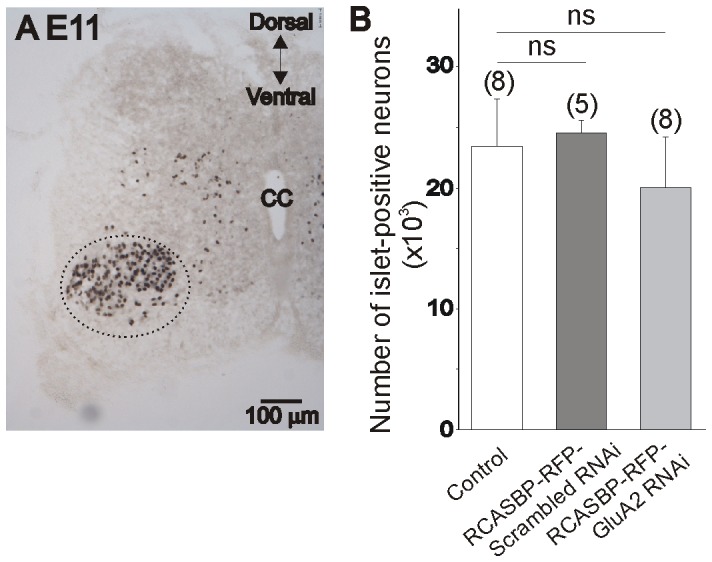
Effect of GluA2 RNAi on motoneurons survival. ***A*** *) Islet1/2*-staining in the lumbar spinal cord of E11 chicken embryos. Only *Islet1/2*-positive neurons in the motoneurons pool were counted (circled area). *Islet1/2*-positive interneurons located in dorsal and medial portions of the spinal cord were not included in our measurements. ***B*** *)* Total number of *Islet1/2*-positive neurons in the lumbar spinal cord of E11 chicken embryos in control (non-infected) and in RCASBP(B)-RFP-scrambled RNAi or RCASBP(B)-RFP-GluA2 RNAi–infected embryos. Infection of chicken embryos with the RCASBP(B)-RFP-GluA2 RNAi has no overall effect on motoneuron survival when compared with control or RCASBP(B)-RFP-scrambled RNAi-infected embryos.

After establishing that infection of chicken embryos with an RCASBP(B)-RFP-GluA2 siRNA construct results in a significant downregulation of GluA2 expression and an increase in the Ca^2+^ permeability of AMPA receptors, we tested how this manipulation altered the dendritic arborization of developing spinal motoneurons *in ovo*. To answer this question, we traced the dendritic tree of spinal motoneurons from embryos fixed at E6 or E11 following injection of a small amount of DiI into the ischiadic plexus, which innervates the muscles of the hindlimb, lower leg and foot in the chicken embryo [19]. After DiI injection, embryos were returned to an incubator for up to 8 weeks in order to allow the retrograde labeling of the dendritic tree of spinal motoneurons (see Methods). We should note that long term storage of the embryos in fixative (required for the DiI tracing of the dendritic tree) led to the complete elimination of the RFP fluorescence in all infected cells in the spinal cord (results not shown). Therefore, our assessment of the dendritic morphology of labeled motoneurons represents a population study that may include non-infected neurons. Despite this caveat, we believe that our finding should still be valid based on the results in Fig. 1C showing that a majority (≥60%) of spinal motoneurons at any given age were transfected with the RCASBP(B) constructs. Typical examples of the dendritic arborization of DiI traced-motoneurons at E6 and E11 from control (non-infected) and RCASBP(B)-infected chicken embryos are represented in Fig. 3 (A–B). As our previous findings indicate, there is a significant increase in the dendritic complexity of spinal motoneurons between E6 and E11 (Fig. 3A–B, [6]). To quantify changes in the dendritic morphology of motoneurons we traced individual DiI-labeled motoneurons (Fig. 3) using Neurolucida according to our exclusion criteria (see Methods). Quantification of the dendritic arborization of motoneurons at E6 and E11 under different conditions is represented in Fig. 4 and 5, respectively. Comparison of the dendritic morphology of E6 motoneurons in non-infected or RCASBP(B)-RFP-scrambled RNAi-infected embryos did not reveal any significant differences in the dendritic arborization of the motoneurons at this particular age. Thus, no significant changes in the total dendritic arbor/cell, the number of primary dendrites, the number of branch points, and the number of dendritic ends were observed between motoneurons from non-infected or RCASBP(B)-infected embryos at E6 (Fig. 4A–D). Although no significant differences were detected in cell body perimeter (Fig. 4E), a statistically significant difference exists in cell body area following infection of chicken embryos with the RCASBP(B)-RFP-GluA2 siRNA construct when compared with control conditions (Fig. 4F). To investigate whether some changes in the dendritic arborization of the motoneurons may have occurred in specific segments of the dendritic tree, we analyzed changes in the number of dendrites as a function of dendritic order. The total number of dendrites within a segment was plotted as a function of their branch order (Fig. 4G). As represented in Fig. 4G, there were no significant changes in the number of dendrites present up to the 4^th^ dendritic order in motoneurons from non-infected or RCASBP(B)-RFP-scrambled RNAi-infected embryos. These findings suggest that downregulation of GluA2 expression and the formation of Ca^2+^ permeable AMPA receptors has no effect on the dendritic morphology of E6 motoneurons.

To investigate whether transformation of normal Ca^2+^-impermeable AMPA receptors into Ca^2+^-permeable receptors can alter the dendritic arborization of E11 motoneurons, we traced individual DiI-labeled motoneurons at E11 using Neurolucida (Fig. 3B & 5A–G). Our results demonstrate that there were no significant differences in the dendritic morphology of E11 motoneurons from non-infected (control) or RCASBP(B)-RFP-scrambled siRNA-infected embryos as indicated by our measurements of total dendritic arbor/cell, the number of primary dendrites, the number of branch points, and the number of dendritic ends (Fig. 5A–D). Similarly, infection of chicken embryos with the RCASBP(B)-RFP-scrambled siRNA construct had no effect on cell body perimeter or cell body area (Fig. 5E–F). However, infection of chicken embryos with the RCASBP(B)-RFP-GluA2 siRNA construct caused a significant reduction in the dendritic complexity of E11 motoneurons when compared with control or RCASBP(B)-RFP-scrambled siRNA-infected embryos (Fig. 5A–D). Thus, downregulation of GluA2 expression with the RCASBP(B)-RFP-GluA2 siRNA construct resulted in a significant decrease in the total dendritic arbor/cell (Fig. 5A). Changes in the length of the dendritic arbor/cell were not the result of changes in the number of primary dendrites (Fig. 5B). Changes in the length of the dendritic arbor/cell were likely the results of significant changes in the number of branch points, and the number of dendritic ends (Fig. 5C–D). Downregulation of GluA2 expression has no effect on cell body morphology including cell body perimeter and area (Fig. 5E–F). To investigate whether the decrease in the length of the dendritic arbor/cell occurs in specific segments of the dendritic tree, we analyzed changes in the number of dendrites as a function of dendritic order. The total number of dendrites within a segment was plotted as a function of their branch order (Fig. 5G). As represented in Fig. 5G, there is a significant reduction in the number of proximal dendrites (2^nd^ and 3^rd^ order dendritic segments). No changes in the number of dendrites were found in the distal dendritic tree (between the 4^th^ and 8^th^ order dendritic segments). These finding suggests that downregulation of GluA2 expression and the appearance of Ca^2+^-permeable AMPA receptors leads to a significant reduction in the dendritic arborization of developing motoneurons.

Changes in the Ca^2+^ permeability of AMPA receptors may adversely affect motoneuron survival [24]. In order to investigate whether the inhibitory effect of RCASBP(B)-RFP-GluA2 siRNA on the dendritic outgrowth of E11 motoneurons was caused by a decrease of motoneuron survival, we counted the number of *Islet1/2*-positive neurons in the ventral spinal cord using design-based stereology (Fig. 6A–B), see also [15], [18]). *Islet*1/2 is a LIM homeodomain transcription factor that is highly expressed in postmitotic spinal motoneurons [21], [22]. Infection of chicken embryos with the RCASBP(B)-RFP-GluA2 siRNA construct caused no significant changes in the number of *Islet1/2*-positive neurons in the E11 lumbar spinal cord when compared with non-infected or RCASBP(B)-RFP-scrambled siRNA-infected embryos. These results show that downregulation of GluA2 expression and the expression of Ca^2+^-permeable AMPA receptors do not alter motoneuron survival at E11.

**Figure 7:**
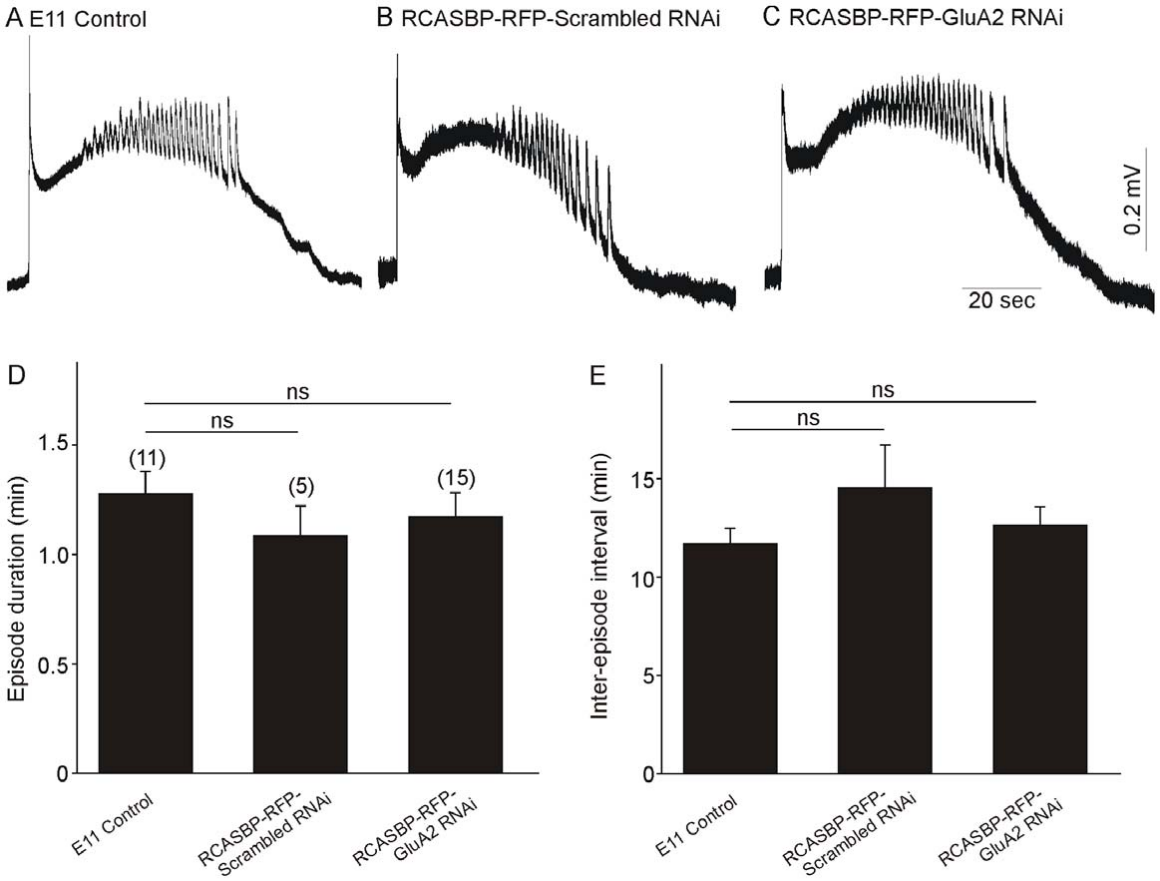
Effect of GluA2 downregulation on the maturation of spontaneous network activity in the chicken spinal cord. ***A–C*** *)* Typical example of an episode of spontaneous activity generated by the spinal cord of control (non-infected, ***A***), RCASBP(B)-RFP-scrambled RNAi (***B***) or RCASBP(B)-RFP-GluA2 RNAi (***C***)-infected embryos at E11. The overall shape of each episode remains the same under each treatment condition. ***D–E***) Downregulation of GluA2 expression did not alter the episode duration *(D)* and inter-episode interval *(* ***E*** *)* in E11 spinal cords.

Previously, we reported that inhibition of spontaneous network activity in the chicken spinal cord can result in a significant reduction in the dendritic arborization of spinal motoneurons between E6 and E11 [18]. In order to determine whether downregulation of GluA2 expression and the appearance of Ca^2+^-permeable AMPA receptors could potentially alter the generation of spontaneous network activity in the chicken spinal cord we recorded the activity generated in the isolated spinal cord at E11. Infection of chicken embryos with the RCASBP(B)-RFP-GluA2 siRNA construct had no noticeable effect on the overall pattern of each episode of network activity in the E11 isolated spinal cord when compared with the pattern of activity generated in control (non-infected) or RCASBP(B)-RFP-scrambled siRNA-infected embryos (Fig. 7A,B,C). Quantification of the effect of RCASBP(B)-RFP-GluA2 siRNA infection on the maturation of network activity in E11 spinal cords indicates that downregulation of GluA2 expression and the appearance of Ca^2+^ permeable AMPA receptors did not disrupt the episode duration (Fig. 7D) or inter-episode interval (Fig. 7E) when compared with the pattern of activity generated in non-infected or RCASBP(B)-scrambled siRNA-infected embryos.

## Discussion

The principal finding of this study is that downregulation of GluA2 expression during a critical period of spinal cord development prevents the maturation of dendritic arborization of spinal motor neurons. During early stages of development, AMPA receptors are highly permeable to Ca^2+^, and this limits the dendritic arborization of spinal motoneuron; subsequently, the expression of GluA2 reduces Ca^2+^ permeability, and dendritic arborization is enhanced. In the chicken spinal cord, the period spanning from E4 to E11 is critical for the development of the lumbar motoneurons and the formation of spinal cord networks. During this period, spinal cord networks initiate the generation of spontaneous electrical activity even before the establishment of synaptic connections between motoneurons and target muscles at E6 [25], [26], [27]. Between E7 and E8, sensory afferents and descending supraspinal inputs begin to establish synaptic contacts with spinal motoneurons, which will ultimately regulate the generation of spinal cord reflexes and locomotor activity, respectively [7], [8]. Changes in the network connectivity of spinal motoneurons are also accompanied by significant changes in their electrophysiological [20], [28], [29] and morphological [6] properties.

Ca^2+^-permeable AMPA receptors are transiently expressed in the chicken spinal cord due primarily to changes in the expression of edited GluA2 subunits [12]. Increased expression of GluA2 subunits renders the AMPA receptors impermeable to Ca^2+^ by E11 [12]. In the present work, we have used the RCASBP(B) system in order to reduce GluA2 expression in developing motoneurons. Infection of chicken embryos when neuronal precursors are diving in the neural tube leads to the uptake and spreading of the viral constructs throughout the spinal cord [15], [30], [31]. Our present results demonstrate that infection of chicken embryos with the RCASBP(B)-RFP-GluA2 siRNA construct resulted in a significant rate of infection of lumbar motoneurons leading to a considerable reduction in GluA2 protein expression. Functionally, downregulation of GluA2 expression caused a significant increase in the Ca^2+^ permeability of AMPA receptors at E11 as demonstrated by our whole cell recordings.

Downregulation of GluA2 expression disrupts the dendritic arborization of spinal motoneurons during a critical period of development. Expression of the RCASBP(B)-RFP- GluA2 siRNA causes a significant reduction in the dendritic length and complexity of the dendritic tree of E11 motoneurons. At this stage of development, GluA2 plays a prominent role in limiting the Ca^2+^-permeability of AMPA receptors. On the contrary, downregulation of GluA2 expression has no effect at E6 since GluA2 subunits are not expressed at age. Analysis of dendritic length as a function of dendritic order also reveals that downregulation of GluA2 mainly affects proximal dendrites and spares more distal dendrites, suggesting that the expression of Ca^2+^-permeable AMPA receptors acts as a stop signal for the growth of specific dendritic segments. We should notice that downregulation of GluA2 expression has no effect on the number of primary dendrites. This is consistent with our previous findings demonstrating that the number of primary dendrites in lumbar motoneurons is already established by E8 [6]. Thus, it appears that the presence of Ca^2+^-permeable AMPA receptors between E6 and E8 has no significant effect in determining the number of primary dendrites. Furthermore, our present findings combined with previous results from our laboratory suggest that different components of the dendritic tree of spinal motoneurons undergo differential regulation by GABA and glutamate neurotransmission [6], [18]. For example, between E6 and E8, the number of primary dendrites appears to be regulated by the depolarizing action of GABA on lumbar motoneurons [18], whereas neurotransmission involving Ca^2+^-permeable AMPA receptors has no effect on the number of primary dendrites at this developmental stage (present results; [6]). On the contrary, proximal dendrites are influenced by both GABA and AMPA receptor activation between E8 and E11 (present results, see also [18]); whereas NMDA receptor activation regulates distal dendrites by E11 [6]. These findings suggest that a multitude of factors regulate the maturation of the dendritic morphology of developing neurons during the establishment of spinal cord networks.

Previous findings have demonstrated that changes in the function of Ca^2+^-permeable AMPA receptors may have opposite effects on dendritic morphology. For example, activation of Ca^2+^-permeable AMPA receptors leads to a significant reduction in the dendritic outgrowth of retinal neurons *in vitro* [32], whereas inhibition of Ca^2+^-permeable AMPA receptors results in a significant increase in the dendritic complexity of chicken motoneurons *in ovo* [6]. These findings support our present results showing that enhanced Ca^2+^ permeability through AMPA receptors limits the dendritic arborization of spinal motoneurons. However, increased expression of Ca^2+^-permeable AMPA receptors leads to a significant increase in the dendritic complexity of matured rat spinal motoneurons [33]. It is likely that these dissimilar effects could have originated from differences in the experimental model used or differences in the developmental stage of the neuronal cells. Alternatively, alterations in the Ca^2+^ permeability of AMPA receptors can potentially influence dendritic arborization as a result of bi-directional changes in intracellular Ca^2+^ and its activation of different downstream signaling pathways or due to changes in the subcellular distribution of AMPA receptors [33], [34].

Considering that intracellular Ca^2+^ dynamics play a significant role in regulating the dendritic arborization of developing neurons [35], [36], it is reasonable to speculate that downregulation of GluA2 expression limits dendritic arborization most likely by altering the Ca^2+^ permeability of AMPA receptors. It is possible, however that downregulation of GluA2 subunits and expression of Ca^2+^-permeable AMPA receptors altered the dendritic morphology of motoneurons by some other Ca^2+^-dependent mechanisms. For example, activation of Ca^2+^-permeable AMPA receptors may lead to membrane depolarization and activation of voltage-gated Ca^2+^ channels. Activation of Ca^2+^ permeable AMPA receptors may also lead to increased activation of NMDA receptors, which are highly permeable to Ca^2+^. We should point out, however, that activation of NMDA receptors appears to enhance dendritic arborization in E11 spinal motoneurons [6]. The role of these processes in regulating the dendritic arborization of developing motoneurons remains to be defined. Further work will also be needed to elucidate the molecular mechanisms involved in the regulation of dendritic arborization by Ca^2+^-permeable AMPA receptors. Time-lapse imaging of dendritic development has demonstrated that dendritic arborization is a highly dynamic process consisting of both the formation of new dendrites and the retraction of already formed dendrites [1], [3]. Thus, activation of Ca^2+^-permeable AMPA receptors could potentially reduce the dendritic complexity of spinal motoneurons by either slowing dendritic outgrowth or stimulating the disassembly of already formed dendrites. Although further experiments are needed to differentiate between these possibilities, it is important to point out the role of intracellular Ca^2+^ in the activity-dependent regulation of dendritic morphology. Various Ca^2+^–dependent intracellular processes involving CaMKII, calcineurin, and Rho GTPases have been implicated in the rapid changes of dendritic length in tectal neurons [37], [38], [39]. Thus, increased Ca^2+^ influx via AMPA receptors may evoke significant changes in dendritic arborization through the activation of various signaling molecules.

Changes in intracellular Ca^2+^ have been implicated in either promoting motoneuron survival or cell death in the spinal cord according to the “Ca^2+^-set point” hypothesis [40]. Furthermore, glutamate receptor activation has been implicated in the glutamate-induced neurotoxicity of spinal motoneurons [41], [42], [43]. Interestingly, our present results indicate that expression of Ca^2+^-permeable AMPA receptors by downregulating GluA2 subunits has no significant effect on motoneuron survival at E11. Thus, it appears that increased Ca^2+^ influx through Ca^2+^-permeable AMPA receptors does not alter motoneuron survival under our experimental conditions. These results are consistent with previous findings demonstrating that pharmacological blockade of AMPA receptors *in ovo* with NBQX does not alter the number of chicken lumbar motoneurons [42], [43]. In E11 motoneurons, increased expression of Ca^2+^-permeable AMPA receptors has no effect on cell body morphology. However, in E8 motoneurons we detected a significant reduction in cell body area in motoneurons infected with the RCASBP(B)-RFP- GluA2 siRNA viral construct. This observation suggests that at different stages of development cell body area may be regulated differently by increased Ca^2+^ influx through AMPA receptors.

Although increased expression of Ca^2+^-permeable AMPA receptors alters the maturation of the dendritic morphology of E11 motoneurons, this event has no effect on the maturation of spontaneous network activity generated by the ventral spinal cord. Spontaneous network activity is an early feature of spinal cord development in the chicken embryo [44], which regulates other developmental processes including motoneuron survival, maturation of neuromuscular junction and ion channel expression [20], [45], [46], [47]. Dendritic arborization regulates the ability of neurons to make appropriate synaptic contacts with postsynaptic targets and to integrate synaptic inputs [2]. However, under our experimental conditions, a reduction in the dendritic arborization of spinal motoneurons by increased expression of Ca^2+^-permeable AMPA receptors has no effect on the normal pattern of spontaneous activity generated by spinal cord networks. We have observed a similar effect following disruption of the dendritic arborization of spinal motoneurons following the inhibition of GABA-driven activity in the chicken spinal cord [18]. Previous results have demonstrated that compensatory changes in electrical excitability of motoneurons regulate the recovery of spontaneous network activity following perturbations of synaptic neurotransmission in the chicken spinal cord [48], [49]. Thus, it is possible that changes in the dendritic arborization of motoneurons expressing Ca^2+^-permeable AMPA receptors evoke compensatory changes in the electrical properties of the motoneurons in order to maintain similar network output in the spinal cord. Another factor that may contribute to maintain the same level of network output in the face of significant changes in dendritic morphology is synaptic scaling [4]. For example, manipulation of dendritic length evokes inversely proportional changes of synaptic strength in hippocampal neurons. An increase in dendritic length following overexpression of β-catenin or increased neuronal activity with extracellular K^+^ results in a reduction in the amplitude of mEPSPs, whereas the opposite effect on mEPSPs was observed following a decrease in dendritic length [4]. Thus, changes in dendritic arborization can potentially result in coordinated changes in synaptic strengths in order to maintain the same level of network output.
